# Bloodlettings in Hemochromatosis Result in Increased Blood Lead (Pb) Concentrations

**DOI:** 10.1007/s12011-022-03424-y

**Published:** 2022-09-27

**Authors:** Mazyar Yazdani, Sonia Distante, Lars Mørkrid, Rune J. Ulvik, Bjørn J. Bolann

**Affiliations:** 1 Department of Medical Biochemistry, Oslo University Hospital, Oslo, Norway; 2 Department of Clinical Medicine, University of Oslo, Oslo, Norway; 3 Department of Clinical Science, University of Bergen, Bergen, Norway; 4 Department of Medicine, Section of Hematology, Haukeland University Hospital, Bergen, Norway; 5 Department of Medical Biochemistry and Pharmacology, Haukeland University Hospital, Bergen, Norway

**Keywords:** Hemochromatosis, Bloodletting, Trace elements, Lead, Mercury, Cadmium

## Abstract

Hemochromatosis is a hereditary disorder, most often associated with mutations of the HFE (High FErrum) gene. If left untreated, it can result in severe parenchymal iron accumulation. Bloodletting is the mainstay treatment. We have previously shown that treatment of hemochromatosis by repeated bloodlettings may induce changes in the serum levels of several trace elements. The aim of this work was to evaluate if whole blood concentrations of the environmental pollutants lead (Pb), mercury (Hg), and cadmium (Cd) could be affected by bloodlettings. We recruited 28 patients and 21 healthy individuals (control group). Whole blood and urine levels of Pb, Hg, and Cd were measured before the start and after the completion of treatment using inductively coupled plasma mass spectrometry, together with serum iron and liver function tests. Concentrations of blood Pb, but not Hg or Cd, were significantly increased after treatment. The increase in Pb was higher in C282Y homozygous patients than in the other patients, and it was positively correlated with the serum concentration of alkaline phosphatase. Bloodlettings in hemochromatosis result in an increase in the blood concentration of Pb. Augmented absorption due to iron loss or Pb mobilization from bone may contribute to the higher blood Pb level.

**Supplementary Information:**

The online version contains supplementary material available at 10.1007/s12011-022-03424-y.

## Introduction

Hemochromatosis is an inherited iron overload disorder characterized by excessive absorption of iron caused by deficiency of hepcidin [1]. It is the most common hereditary disorder in the Nordic countries [2]. Permanently increased iron (Fe) uptake from the gut results in iron accumulation and overload, leading to severe parenchymal damage, particularly in the liver and the heart, joints, and other organs [3]. If left untreated, the disorder may result in a lethal outcome.

Different types of hereditary hemochromatosis are defined by the specific mutation involved [3]. In most cases, the disorder is associated with mutations of the High FErrum (HFE) gene. Homozygosity for C282Y is the most prevalent variant in patients with symptoms, although other variants like H63D homozygosity or compound heterozygosity C282Y/H63D may contribute to disease manifestations [4]. Routine treatment involves bloodlettings of 450–500 mL weekly, up to 20–40 times, to remove excess iron from the body. After normalization of iron parameters, patients need maintenance blood lettings throughout their lives [5, 6].

The pathogenetic mechanisms of hemochromatosis are not fully understood. The genetic mutations have variable phenotypic penetrance, and the development of clinical symptoms seems to be modulated by yet unknown factors [6, 7]. Although it is not a gender-specific disease, the symptoms occur more often in male patients [8]. In women, clinical symptoms are usually presented later because of blood loss experienced with menstruation and childbirth.

Multiple interrelationships between serum levels of iron and various trace elements have been demonstrated [9, 10]. Disturbances in iron metabolism may affect the metabolism of metals other than iron [9, 11–14]. Iron-binding proteins like transferrin and ferritin can bind other metals in addition to iron [15–20].

Barton et al. [21] found that hemochromatosis patients, especially homozygotes, absorb increased quantities of lead. In contrast, Åkesson [12] demonstrated that blood concentrations of cadmium, but not lead, were significantly higher in bloodletted hemochromatosis patients than in paired controls. The reason for this discrepancy is not clear. Beyond these and our previous study [22], we have found no other report on the effects of bloodlettings on trace element status in hemochromatosis patients.

The aim of this study was to see if bloodlettings in hemochromatosis patients affect whole blood concentrations of the environmental pollutants lead (Pb), mercury (Hg), and cadmium (Cd). We recruited untreated patients and compared pre-phlebotomy blood concentrations with post-phlebotomy values in the same individuals using a prospective, pairwise design. In addition, a group of healthy persons without hemochromatosis and not subject to bloodlettings were included as controls.

## Materials and Methods

### Reagents

Seronorm™ Trace Elements Whole Blood controls were obtained from SERO AS (Billingstad, Norway). HNO_3_ and Triton® X-100 were purchased from Merck (KGaA, Darmstadt, Germany) and gold from PerkinElmer Inc. (Shelton, Connecticut, USA).

### Subject Selection

**Fig. 1:**
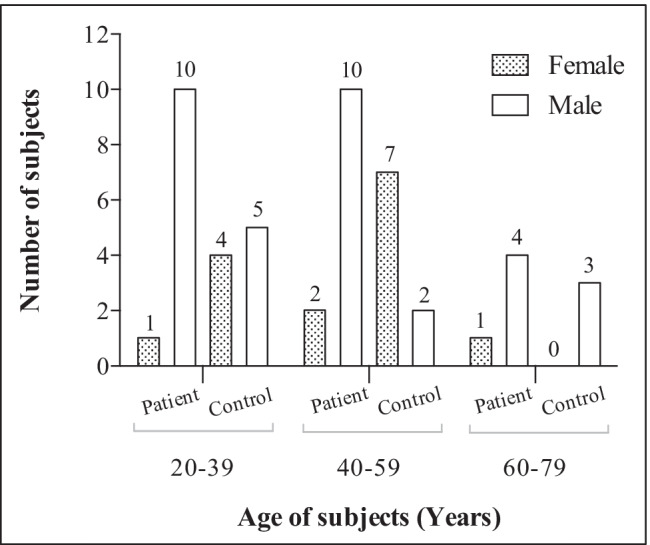
Age and sex distribution of subjects

Twenty-eight patients and twenty-one healthy individuals (controls) were recruited (Fig. 1). For prospective pairwise comparisons, samples from the patients were analyzed before the start and after the completion of treatment (bloodlettings) aimed at normalizing serum iron parameters. Exclusion criteria were age less than 18 years, other bloodletting or transfusion within the last 3 months, concurrent disease, pregnancy, installed osteosynthesis materials (e.g., after fractures), or other metal items.

### Informed Consent

The study was approved by the Regional Committee for Medical and Health Research Ethics, Western Norway (REC no. 220.05). Informed consent was obtained from patients referred by their doctors to the hemochromatosis outpatients’ clinics at Haukeland University Hospital, Bergen and Oslo University Hospital, Oslo, Norway, and from control persons.

### Blood and Urine Collection

All the patients were treated with venesection of 450 mL blood either weekly or every alternate week until normalization of iron parameters, which could take up to 24 bloodlettings. Blood samples were collected for trace element and hematological analyses and serum samples for iron status and clinical chemistry measurements. Urine samples were also collected for most of the patients. Clinical chemistry and hematological analyses were done as described previously [22]. Transferrin iron saturation (Tfsat) was calculated as the molar ratio between serum iron and total iron binding capacity (TIBC).

### Trace Element Analysis and Analytical Quality Control

Whole blood samples were collected on BD Vacutainer K2 EDTA Trace Elements (Puls Norge, Oslo). Prior to analysis, the samples were diluted 1:25 with 0.33% v/v (volume per volume) HNO_3_, 0.1% v/v Triton® X-100, and 0.5 ppm gold. Trace elements were measured by inductively-coupled plasma mass spectrometry (ICP-MS) on Perkin Elmer ELAN DRC-e (PerkinElmer, Toronto, Canada) using a standard mode [22, 23]. The lower limits of quantification (LQ) were defined as five times the within-day analytical standard deviation, as determined by 20 measurements in a sample pool—this gives a theoretical coefficient of variation of 20% for LQ [24]. LQ for Pb, Hg, and Cd was 0.01 μmol/L, 4.7 nmol/L, and 1.6 nmol/L, respectively.

The between-run analytical coefficients of variation for Pb, Hg, and Cd, as determined in Seronorm Trace Elements Wholeblood Level 1, were 3%, 10%, and 10%, respectively. All analyses complied with assigned values for Seronorm Trace Elements Wholeblood Level 1, 2, and 3. Urine metal concentrations are given as the molar ratio urine metal/urine creatinine concentration [25].

### Statistical Analysis

The concentrations of trace elements and other variables before the start and after the completion of treatment were compared using related samples Wilcoxon signed-rank test. All paired analyses were done in the same analytical run. Correlation coefficients were calculated as Spearman’s rho. Other comparisons were done as stated to the text and tables. In our calculations, the genotypes were dichotomized to C282Y homozygote vs. all other genotypes. All statistical analyses were performed with IBM SPSS Statistics Version 25 (IBM Corp., Armonk, NY). GraphPad Prism 6.0 for Mac (GraphPad Software, San Diego, CA, USA) was used for preparing the figures.

## Results

Figure 1 and Supplementary Table 1 present the subject demographics. As could be expected [8], the number of male patients (*n* = 24) was higher than that of female patients (*n* = 4), in contrast to the control group (males = 10; females = 11). The age group of 60–79 years presented the lowest number of participants for both the patient group (*n* = 5) and the control group (*n* = 3). The group-wise scatter plots of blood Pb, Cd, and Hg by age are shown in Supplementary Fig. 1.

**Fig. 2:**
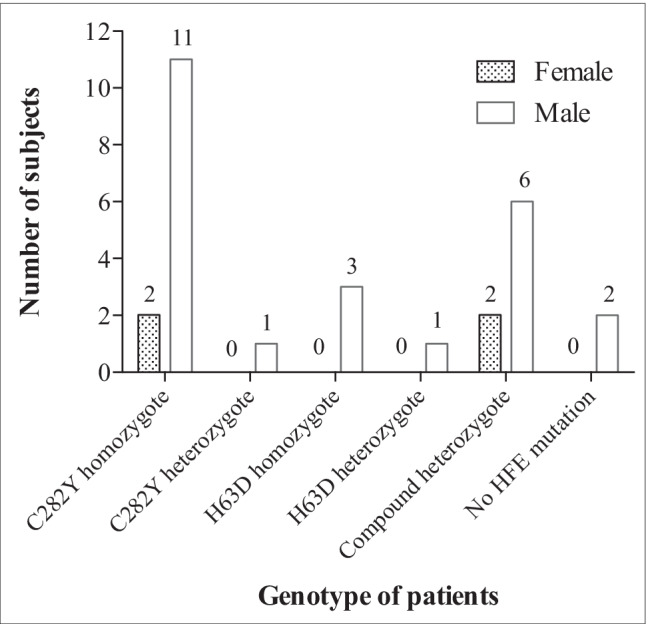
Distribution of genotypes by gender

Genotypes of the patients are shown in Fig. 2. Half of the patients had C282Y mutation (homozygote = 13; heterozygote = 1). Two male patients had raised iron parameters but no HFE mutation; in these cases, the clinical diagnosis of hemochromatosis with iron overload was confirmed by tolerance to repeated therapeutic bloodlettings.

Table 1 summarizes the correlations among iron status (as represented by ferritin), hemoglobin (Hb), and clinical chemistry variables in the patients before and after the treatment, as well as those in the control group. In patients not yet subjected to bloodletting, ferritin levels were significantly correlated with Hb (*r* = 0.439), gamma-glutamyltransferase (GGT, *r* = 0.610), and alanine aminotransferase (ALT, *r* = 0.791). After bloodlettings, a significant correlation to ferritin was found for only creatinine (*r* =  − 0.407). In the control group, there were significant positive correlations of ferritin with three parameters: Hb (*r* = 0.515), GGT (*r* = 0.468), and alkaline phosphatase (ALP, *r* = 0.593).

**Table 1:** Correlations among variables. Correlation (Spearman’s rho coefficients) of serum ferritin with Hb and liver function test parameters in patients before and after bloodletting and in the control group

	Bloodletted?	n	Hb	Creatinine	GGT	ALT	ALP
Ferritin	No	28	0.439*	0.477	0.610**	0.791**	0.351
	Yes	28	0.294	− 0.407*	0.356	− 0.262	− 0.192
	Control	21	0.515*	0.168	0.468*	0.410	0.593**

^*^p < 0.05

^**^p < 0.01

The significant *p* values have been highlighted in bold fonts

Correlations of the abovementioned variables with trace elements are shown in Table 2. Before bloodlettings, significantly positive associations were observed between Pb and the liver enzymes GGT (*r* = 0.477) and ALP (*r* = 0.388). Of these, only the correlation between Pb and ALP (*r* = 0.465) persisted after bloodlettings. There was no significant correlation between Pb and iron status. There were negative correlations of Hg levels with some iron parameters, and Hg levels were significantly correlated with serum creatinine levels in all the groups. Cd was negatively correlated with TIBC both before (*r* =  − 0.593) and after (*r* =  − 0.615) the bloodlettings. These and other correlations are shown in Table 2.

**Table 2:** Correlations among variables. Correlation (Spearman’s rho coefficients) of trace metal concentrations with Hb, liver function test parameters, and iron profiles in patients before and after bloodletting and in the control group

	Bloodletted?	*n*	Iron	TIBC	Ferritin	Hb	Tfsat	Creat	GGT	ALT	ALP
Pb	No	28	− 0.269	0.120	0.195	0.329	− 0.308	0.095	0.477*	0.194	0.388*
	Yes	28	− 0.028	− 0.230	− 0.008	0.450*	− 0.070	− 0.092	0.152	− 0.013	0.465*
	Control	20	0.307	0.161	− 0.037	0.145	0.300	0.120	0.397	0.451*	0.217
Hg^1^	No	22	− 0.510*	0.232	0.016	0.249	− 0.481*	0.431*	0.401	0.111	0.032
	Yes	22	0.108	0.202	− 0.445*	0.056	− 0.014	0.614**	0.453*	− 0.006	0.231
	Control	16	− 0.031	− 0.187	0.394	0.436	0.124	0.538*	0.438	0.530*	0.436
Cd^1^	No	18	− 0.119	− 0.593**	0.138	0.287	0.007	0.043	0.081	0.050	0.542*
	Yes	20	− 0.255	− 0.615**	− 0.078	− 0.015	− 0.070	− 0.001	− 0.109	0.011	0.376
	Control	8	0.310	0.108	− 0.357	0.190	0.286	− 0.071	0.263	0.095	0.095

^*^p < 0.05

^**^p < 0.01

^1^Results below limit of quantification excluded

The significant *p* values have been highlighted in bold fonts

Table 3 shows the correlations among trace elements. In the control group, the levels of Pb were significantly associated with those of Cd in the blood (*r* = 0.922) and urine (*r* = 0.693); however, in the patient group, this association was found only in the urine after treatment (*r* = 0.674). Blood Pb was also correlated with Hg in untreated patients (*r* = 0.514) and in controls (*r* = 0.688).

**Table 3:** Correlations among variables**.** Correlation (Spearman’s rho coefficients) between trace metal concentrations in patients before and after bloodletting and in the control group

		Bloodletted?	Hg (*n*)^†^	Cd (*n*)^†^
Blood	Pb	No	0.514* (22)	0.407(18)
		Yes	0.182 (22)	0.448 (20)
		Control	0.688** (16)	0.922** (8)
	Hg	No		0.159 (15)
		Yes		− 0.388 (16)
		Control		0.760 (7)
Urine	Pb	No	NA	0.454 (15)
		Yes	NA	0.674** (16)
		Control	0.394 (10)	0.693* (19)
	Hg	No		NA (2)
		Yes		NA (1)
		Control		0.083 (9)

^*^p < 0.05

^**^p < 0.01

^†^Spearman’s rho coefficients (*n*)

*NA*, not available

Results below limit of quantification excluded

The significant *p* values have been highlighted in bold fonts

The results from paired comparisons of iron status, Hb levels, and clinical chemistry variables in patient and control groups are provided in Table 4 (*p* values in Supplementary Table 2). Iron status (iron, TIBC, ferritin, and Tfsat) of untreated patients was significantly different from controls, and bloodletting changed it significantly in the patient group. There was a significant difference between untreated patients and controls for iron status, Hb, and ALT. GGT levels differed significantly between controls and patients, both before and after treatment. As shown in Table 5, Pb in blood increased by 26% (median, *p* < 0.001) after the bloodlettings. In addition, Pb levels were significantly higher in the treated patients than in the control group (*p* values in Supplementary Table 3).

**Table 4:**
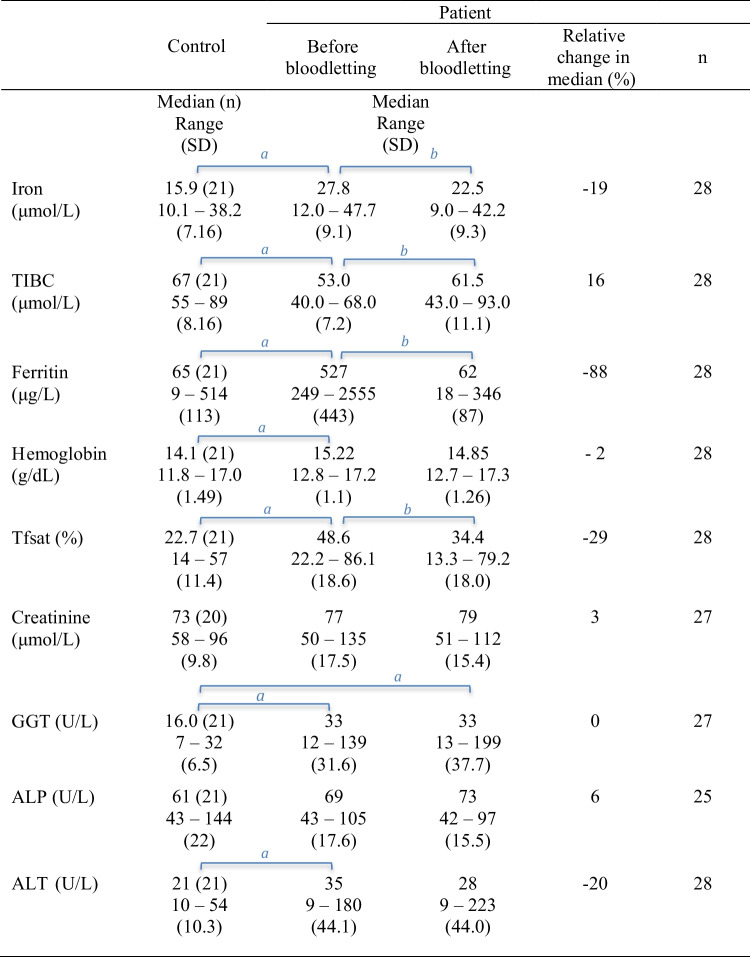
Paired comparisons of serum iron profiles, Hb, serum creatinine, and liver function test parameters in patients and controls

^*a*^Independent samples Mann–Whitney *U* test for inter-group comparisons

^*b*^Related samples Wilcoxon signed-rank test for paired comparisons

Letters (^*a*^ or ^*b*^) above groups denote significant differences. Supplementary Table 2 provides the *p* values

**Table 5:**
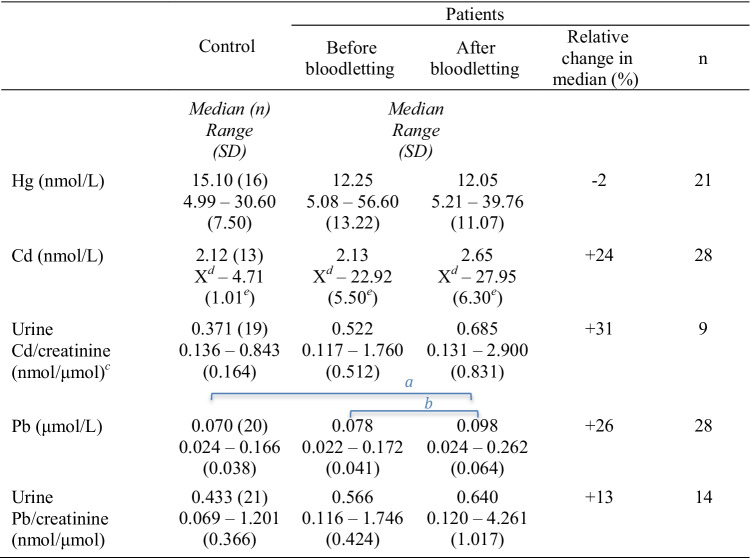
Paired comparisons of trace metal concentrations in patients and controls

^*a*^Independent samples Mann–Whitney *U* test for inter-group comparisons

^*b*^Related samples Wilcoxon signed-rank test for paired comparisons

^*c*^Values < LQ in u-Cd excluded

^*d*^X = Result below LQ

^e^Values below LQ excluded

Letters (^*a*^ or ^*b*^) above groups denotes significant difference. Supplementary Table 2 provides the *p* values

**Table 6:** Increase in Pb concentrations: relationships with pre-treatment variables. Increase in blood lead concentrations in subjects with different hemochromatosis genotypes (μmol/L)

	Median	Range	*n*
C282Y homozygote	0.034	− 0.004 to 0.114	13
C282Y heterozygote	0.008		1
H63D homozygote	0.002	− 0.012 to 0.011	3
H63D heterozygote	0.000		1
Compound heterozygote	0.024	− 0.007 to 0.063	8
No HFE mutation	0.026	− 0.010 to 0.062	2
Total	0.029	− 0.012 to 0.114	28

**Fig. 3:**
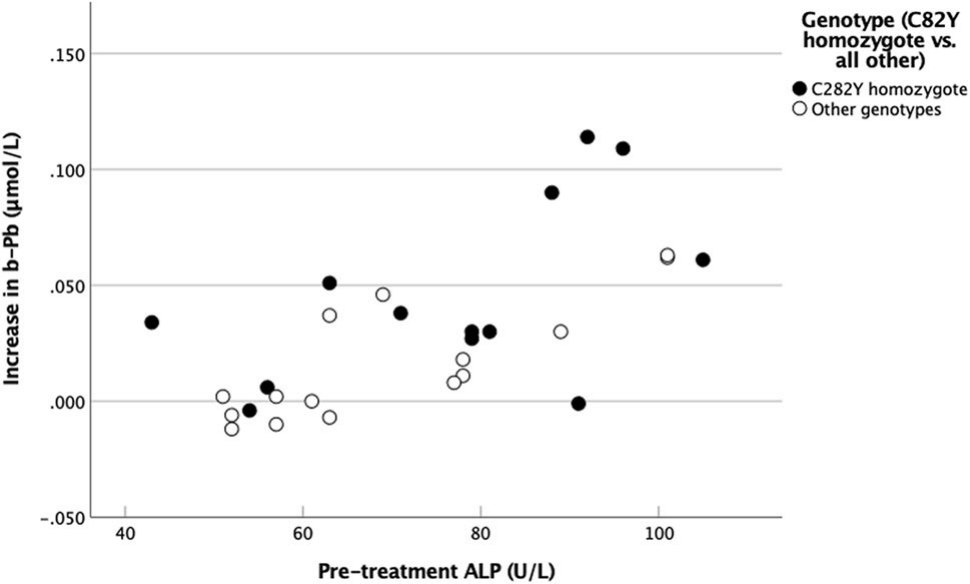
The relationship of Pb levels with pre-treatment ALP levels in blood

Table 6 shows the increase in blood Pb by genotypes. The increase was higher in C282Y homozygote patients than in patients with other genotypes (Table 7, *p* = 0.048). Among other pre-treatment variables, only ALP was significantly correlated with the increase in blood Pb (*p* < 0.001, Table 7). The relation between ALP and the increase in Pb is shown in Fig. 3.

**Table 7:** Increase in Pb concentrations: relationships with pre-treatment variables. Correlation (Spearman’s rho coefficients) between increase in Pb and serum iron profiles, Hb, serum creatinine, liver function test parameters, and C282Y homozygote genotype

		Ferritin	Iron	TIBC	Tfsat	Hb	GGT	ALT	ALP	Creat	Genotype^*a*^
Increase in b-Pb	Spearman’s rho	0.103	0.260	− 0.322	0.321	0.143	− 0.019	− 0.062	0.662	− 0.145	0.337
	*p*	0.603	0.182	0.095	0.096	0.468	0.922	0.754	** < 0.001**	0.462	**0.048**
	n	28	28	28	28	28	28	28	28	28	28

^*a*^C282Y homozygote vs. all others

The significant *p* values have been highlighted in bold fonts

## Discussion

### Toxic Metals

In this work, we demonstrate that bloodlettings performed to eliminate iron overload in hemochromatosis patients increase the concentration of Pb in blood. The concurrent increase in the concentration of Cd was not statistically significant, whereas Hg levels were not affected by the bloodlettings (Table 5 and Supplementary Table 3). The findings are in line with those of our previous study, which showed that several other trace elements in the serum might be influenced by bloodlettings [22]. Åkesson et al. [12] observed that both Cd and Pb levels increased after phlebotomy in hemochromatosis patients; however, the increase was statistically significant only for Cd. The discrepancy between Åkesson’s and our results might have been caused by the limited number of participants in both studies. In our study, the number of male patients (*n* = 24) was higher than that of female patients (*n* = 4), such uneven gender distribution has been previously reported [8].

To our knowledge, no other publication has attempted to show the effects of bloodletting therapy on micromineral homeostasis in hemochromatosis patients. However, several investigations have revealed associations between iron and various microelements [9, 10, 26]. The observation that the blood concentration of Pb was influenced by bloodlettings (Table 5) may be partly explained by the associations between HFE mutations and blood Pb levels [27–29]. It has been reported that hemochromatosis patients, especially homozygotes, absorb increased quantities of Pb compared to normal persons [21]. In line with this, our patients with the homozygote genotype C282Y mutation presented a higher increase in blood Pb concentration than the other patients (Tables 6 and 7). The adverse health effects of Pb, even at very low blood levels, are well-known [30–34]. Although the concentrations of Pb reported in this study were low, adverse effects of increased Pb absorption or mobilization cannot be ruled out.

Hg was detected in the blood samples of all participants. In contrast to Pb, the Hg concentration was not affected by the treatment (Table 5 and Supplementary Table 3). Due to global contamination, Hg is generally present in humans. Fish and seafood are the leading sources of Hg exposure [35], especially in societies with high consumption, like the Nordic countries [36]. The median Cd concentration was higher after the bloodlettings than before in both blood and urine (Table 5 and Supplementary Table 3), although the difference was not statistically significant. However, Cd concentration in the patients was highly dispersed, much more than in the control persons. There was a significant correlation between Pb and Hg levels in the blood (Table 3). Pb was also correlated with Cd in the controls (Table 3). Correlations among Hg, Pb, and Cd in the general population have been assumed to be due to common exposure sources and accumulation in the body [37].

Cd was negatively correlated with TIBC in untreated and treated individuals (Table 2). A similar negative correlation between blood Cd and iron status was reported previously [38–41]. Cd levels may increase under iron overload conditions [42].

### Liver Function Tests

Pb concentration before the bloodlettings was correlated with ALP and GGT levels (Table 2). Elevated levels of liver enzymes may be an early sign of this organ’s injury due to iron accumulation. In agreement with a previous study [4], liver enzyme levels were correlated with the ferritin level (Table 1). Moreover, ALP is related to bone disease, and Pb is known to accumulate in bones [43]. The strong correlation of pre-treatment ALP with both Pb (Table 2) and the subsequent increase in Pb levels (Table 7 and Fig. 3) may suggest that Pb is mobilized from the bones into the blood. Notably, ALP levels were not significantly altered by the bloodlettings (Table 4 and Supplementary Table 2). Interestingly, the levels of ALT, another liver enzyme, were significantly correlated with Pb levels in control persons only (Table 2). A corresponding relationship of ALT with Hg was detected (Table 2). Hg is the trace element showing a significant correlation with Pb levels in the blood (Table 3). In the control group, Pb was also correlated with Cd (Table 3). However, this did not result in a significant correlation of ALT with Cd (Table 2), possibly due to the low number of samples (*n* = 8).

Hg concentration was correlated with ALT levels in only the control group (Table 2). In a cohort study of healthy premenopausal women (*n* = 259) [44], the associations of low exposure levels to Hg and some functional biomarkers of the liver and kidney were revealed, similar to our results for GGT, ALT, and creatinine. For Cd, our study found a correlation with ALP levels (Table 2). It could be an early indication of liver injury in untreated patients [45, 46]. Cd exists in tobacco, and thus, humans are exposed to Cd through tobacco smoke [47], but we do not have complete data on the smoking status of the recruited individuals. In serum, this environmental and industrial pollutant is bound to alfa-2-macroglobulin and albumin [48]. Cd chiefly accumulates in the liver and kidneys of exposed individuals.

Creatinine concentration was the only parameter in the present study that was correlated with Hg concentration in all subjects (Table 2), although no significant difference between groups was found as a result of the bloodlettings (Table 4 and Supplementary Table 2). The kidneys are one of the main targets of Hg deposition and subsequent toxicity [49], whereas creatinine is frequently used as a biomarker of renal function [50]. Pb concentration was not significantly correlated with creatinine levels (Table 2).

### Iron Metabolism

Several organs, including the duodenum, liver, and bone marrow, are involved in regulating iron metabolism [8]. Iron and other elements compete for biomolecular binding sites for transportation, e.g., DMT1, transferrin, and ferritin [17, 51, 52]. The induced duodenal absorption of iron (e.g., by bloodletting) may affect the fate of some other metals such as Pb, Cd, and Co [53–55]. In line with this, while the repeated bloodlettings removed iron from the body and normalized the iron status (Table 4), the absorption or mobilization of Pb increased (Table 5).

The strength of this work is its design, which allows pairwise comparisons of the included patients. Weaknesses include the limited number of participants for each genotype, the imbalance between genders, the absence of data on diet, medications, and lifestyle (e.g., drinking and smoking habits) of the participants, and the unavailability of data for urine Hg levels.

## Conclusion

The present and previous works demonstrate that repeated bloodlettings in hemochromatosis affect the blood levels of several metals and not only iron. While serum iron declines, the effect on other metals, if any, is generally an increase in serum or whole blood levels. In the treatment of hemochromatosis, one should be aware that repeated bloodlettings may induce increased absorption or mobilization of toxic metals in the body. Whether this effect is due to increased absorption or redistribution in the body is not clear, and further studies are needed.

## Supplementary Information

Below is the link to the electronic supplementary material.

Supplementary file1 (DOCX 380 KB)

Supplementary file2 (DOCX 29 KB)
