# Supplementary material for: Bloodlettings in Hemochromatosis Result in Increased Blood Lead (Pb) Concentrations
Source: Biol Trace Elem Res. 2022 Sep 27;201(7):3193–201. doi: 10.1007/s12011-022-03424-y (PMC10160177; doi:10.1007/s12011-022-03424-y)
Supplement: Supplementary file 1 — Supplementary file1 (DOCX 380 KB) [file 12011_2022_3424_MOESM1_ESM.docx]

**Supplementary figures**

**Supplementary figure 1.** Effect of age on the distribution of Hg (a), Cd (b), and Pb (c) in blood.

**a.**

**b.**

**c.**

**Supplementary figure 1**
