# Supplementary material for: Bloodlettings in Hemochromatosis Result in Increased Blood Lead (Pb) Concentrations
Source: Biol Trace Elem Res. 2022 Sep 27;201(7):3193–201. doi: 10.1007/s12011-022-03424-y (PMC10160177; doi:10.1007/s12011-022-03424-y)
Supplement: Supplementary file 2 — Supplementary file2 (DOCX 29 KB) [file 12011_2022_3424_MOESM2_ESM.docx]

**Supplementary Table 1.** Age and sex distribution of subjects recruited for the study.

| **Age (Years)** | **20 – 39** | **40 – 59** | **60 – 79** | **Total** |
| --- | --- | --- | --- | --- |
| ***Patients*** |  |  |  |  |
| Female | 1 | 2 | 1 | 4 |
| Male | 10 | 10 | 4 | 24 |
| **Total** | 11 | 12 | 5 | 28 |
| ***Controls*** |  |  |  |  |
| Female | 4 | 7 | 0 | 11 |
| Male | 5 | 2 | 3 | 10 |
| **Total** | 9 | 9 | 3 | 21 |

The date of sampling and analysis:

First sample: From April 2007 to October 2011

Last sample: From January 2008 to August 2012

Controls: From October 2006 to June 2007

# Supplementary Table 2. Inter-group comparisons (*p*-values) of biochemical variables for iron status, haemoglobin, and clinical chemistry tests in patient and control groups.

|  | **Before bloodletting vs control*^a^*** | **After bloodletting vs bloodletted *^b^*** | **Bloodletted vs control*^a^*** |
| --- | --- | --- | --- |
| **Iron (µmol/L)** | **<0.001** | **0.035** | 0.08 |
| **TIBC (µmol/L)** | **<0.001** | **<0.001** | 0.093 |
| **Ferritin (µg/L)** | **<0.001** | **<0.001** | 0.724 |
| **Haemoglobin (g/dL)** | **0.003** | 0.104 | 0.069 |
| **Tfsat (%)** | **<0.001** | **0.002** | 0.052 |
| **Creatinine (µmol/L)** | 0.229 | 0.990 | 0.155 |
| **GGT (U/L)** | **< 0.001** | 0.187 | **0.001** |
| **ALP (U/L)** | 0.099 | 0.330 | 0.114 |
| **ALT (U/L)** | **0.004** | 0.059 | 0.050 |

*^a^* Independent samples Mann-Whitney U test

*^b^* Related samples Wilcoxon signed-rank test

The significant *p*-values have been highlighted in bold fonts

**Supplementary Table 3.** Inter-group comparisons (*p*-values) of the concentrations of trace elements in patient and control groups.

|  | **Before bloodletting vs control*^a^*** | **After bloodletting vs control*^a^*** | **Before bloodletting vs bloodletted*^b^*** |
| --- | --- | --- | --- |
| **Blood Hg (nmol/L)** | 0.693 | 0.510 | 0.664 |
| **Blood Cd (nmol/L)** | 0.367 | 0.128 | 0.202 |
| **Urine Cd/creatinine (nmol/µmol)*^c^*** | 0.391 | 0.443 | 0.314 |
| **Blood Pb (µmol/L)** | 0.140 | **0.004** | **<0.001** |
| **Urine Pb/creatinine (nmol/µmol)** | 0.478 | 0.341 | 0.551 |

*^a^* Independent samples Mann-Whitney U test

*^b^* Related samples Wilcoxon signed-rank test

*^c^* Samples with urine concentration below LQ excluded

The significant *p*-values have been highlighted in bold fonts
